# Spatiotemporal Patterns of Microplastic Distribution in Surface Water and Nearshore Sediments of a Large Tropical Lake: Evidence From Lake Toba, Indonesia

**DOI:** 10.1155/sci5/3525274

**Published:** 2026-10-28

**Authors:** Ahmad Muhtadi, Rusdi Leidonald, Aldiano Rahmadya, Prawira Atmaja Tampubolon, Nur Rohim, Muhammad Reza Cordova, Mohd Yusoff Ishak

**Affiliations:** ^1^ Department of Aquatic Resources Management Faculty of Agriculture Universitas Sumatera Utara Medan 20155 North Sumatra Indonesia usu.ac.id; ^2^ Research Centre for Limnology and Water Resources National Research and Innovation Agency Jl. Raya Jakarta-Bogor Km. 46. Cibinong Bogor 16911 West Java Indonesia brin.go.id; ^3^ Biota Systems Research Center National Research and Innovation Agency Jl. Raya Jakarta-Bogor Km. 46. Cibinong Bogor 16911 West Java Indonesia brin.go.id; ^4^ Research Centre for Oceanography National Research and Innovation Agency Jakarta Indonesia brin.go.id; ^5^ Department of Environment Faculty of Forestry and Environment Universiti Putra Malaysia Seri Kembangan Malaysia upm.edu.my

**Keywords:** Lake Toba, large tropical lake, microplastics, nearshore sediment, seasonal variation, spatial distribution

## Abstract

Microplastic (MP) pollution is increasingly documented in freshwater ecosystems, yet lake‐scale evidence from large tropical lakes remains limited. We assessed MPs in surface water and nearshore sediments at 21 stations across Lake Toba, Indonesia, during the dry and rainy seasons. Mean MP abundance was higher in the rainy season than in the dry season in both surface water (1.461 ± 0.836 vs. 0.288 ± 0.110 particles m^−3^) and sediment (3.14 ± 0.59 vs. 1.26 ± 0.31 particles g^−1^ dry weight). Fragments and particles measuring 1000–2000 μm were dominant, while black and blue particles jointly represented 67%–68% of the observed MPs. Surface‐water abundance was highest in the northern sector, whereas nearshore sediment abundance was higher in the southern sector. These contrasting patterns are consistent with spatial redistribution and retention within the lake, although transport pathways and sediment‐focusing processes were not measured directly. Aquaculture, fishing, tourism, and domestic activities are therefore discussed as potential rather than confirmed sources. This study provides the first seasonally resolved, lake‐wide baseline integrating surface water and nearshore sediments in Lake Toba and identifies priorities for source‐specific monitoring and hydrodynamic investigation.

## 1. Introduction

With over 14 million tonnes of plastic entering aquatic systems annually [1], plastic pollution has become a critical global environmental challenge. The rapid growth in plastic production, from approximately 1.5 million tonnes per year in the 1950s to over 400 million tonnes in 2022 [2–4], combined with its low cost, durability, and versatility [5], has resulted in substantial plastic leakage into aquatic environments. Once introduced, plastics persist and progressively fragment into microplastics (MPs), threatening aquatic ecosystems, biodiversity, and human health [6–8].

MPs are generally defined as synthetic polymer particles smaller than 5 mm [9–11]. The operational size range recovered in a study nevertheless depends on the sampling and analytical methods. Here, the 300 μm mesh used for surface‐water sampling established a lower operational detection limit of approximately 300 μm; consequently, smaller MPs and nanoplastics were outside the scope of the present assessment. MPs are environmentally relevant because their persistence and size facilitate transport, ingestion by aquatic organisms, and interactions with other contaminants [12–14].

Research on MPs in lakes has expanded substantially, including recent syntheses of their sources, distribution, and environmental effects [15, 16]. However, spatially extensive and seasonally resolved studies that jointly examine water and sediment remain comparatively uncommon for large tropical lakes. Within‐lake circulation, particle properties, biofouling, aggregation, and depositional conditions may decouple concentrations in surface water from those in sediments. Such mechanisms can be evaluated only indirectly from spatial surveys unless they are supported by hydrodynamic measurements, sediment cores, or particle‐transport modeling.

In Indonesia, MP research has primarily focused on marine and estuarine environments [17–20], whereas studies on lake systems remain sparse, with most limited to a few sites, such as Lake Singkarak [21], Lake Beratan [22], and urban lakes in the Jakarta region [23]. Despite its ecological significance, Lake Toba, the largest volcanic lake in Southeast Asia, has not previously been assessed for MP distribution or sediment focusing, representing a critical knowledge gap in large‐lake MP research in tropical regions.

This study assessed the spatial and seasonal distribution of MPs in the surface water and nearshore sediments of Lake Toba. We tested two focused hypotheses: (1) MP abundance differs among lake sectors in both environmental matrices and (2) MP abundance differs between the dry and rainy seasons. We also evaluated whether the observed water‐sediment patterns were consistent with potential anthropogenic inputs and within‐lake redistribution. Because source apportionment, hydrodynamic circulation, and deep‐basin deposition were not measured directly, interpretations of sources and transport mechanisms are treated as hypotheses rather than demonstrated processes.

## 2. Materials and Methods

### 2.1. Study Area and Sampling Strategy

Lake Toba, located in North Sumatra, Indonesia, is the largest volcanic lake in the world and the largest lake in Southeast Asia [24]. It has a surface area of approximately 1124 km^2^ and has an estimated volume of 256.2 km^3^ [25] and supports capture fisheries, floating net‐cage aquaculture, transportation, raw‐water supply, hydropower generation, and tourism enterprises [26–28]. The lake is also designated as a National Priority Tourism Destination [29, 30].

MP sampling was conducted at 21 stations distributed across the northern, eastern, southern, and western sectors of Lake Toba, including sites around Samosir Island and the Sumatra mainland (Figure 1). Stations were selected purposively to provide lake‐scale coverage of contrasting land uses and hydrological settings. The design included intensive floating net‐cage aquaculture areas (Stations 2, 3, and 13), principal tourism areas (Stations 5, 8, and 21), the largest river inflow (Station 9), the lake’s only surface outflow (Station 7), populated shorelines, and less intensively used comparison sites. Coordinates and site descriptions are provided in Appendix 1. This design supports inference about surface‐water and nearshore‐sediment patterns but does not represent the vertical water column or deep profundal sediments.

**FIGURE 1:**
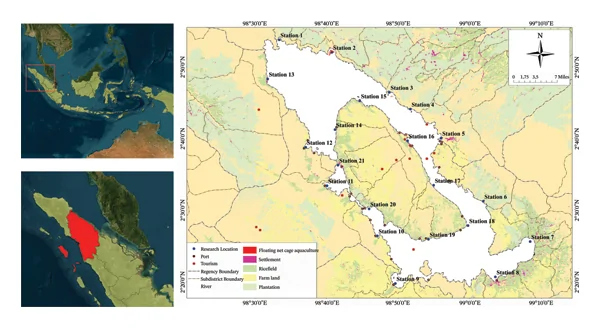
Map of sampling locations in Lake Toba, Indonesia.

Sampling was conducted in August (dry season) and December (rainy season). Surface‐water MPs were collected using a Bongo net with a 60 cm mouth diameter, 300 μm mesh, and calibrated flowmeter. At each station, the net was towed for approximately 20 min at 1–2 knots, filtering 1037.14 ± 94.29 m^3^ of water (range: 942.86–1131.43 m^3^). The net was alternately deployed on either side of the vessel to reduce wake effects and was rinsed after each tow. Samples were transferred to clean 300 mL glass bottles, preserved with 15 mL of 5% formalin, and transported under temperature‐controlled conditions. The 300 μm mesh minimized clogging but excluded smaller MPs from the assessment.

Nearshore sediment was collected at each station from the upper 0–5 cm using a stainless‐steel shovel at water depths of approximately 2–5 m. Approximately 500 g was transferred to a precleaned glass container, preserved with 15 mL of 10% formalin, and transported to the laboratory. These samples characterize littoral or nearshore accumulation only; they were not collected from deep depositional zones and therefore cannot directly demonstrate basin‐scale sediment focusing.

Water and sediment samples were preserved using different concentrations of formalin because of differences in matrix characteristics and organic‐matter content. The reported concentrations of 5% and 10% refer to the formalin working solutions added to the samples rather than to the final formalin concentrations in the sample containers. For the water samples, 15 mL of 5% formalin working solution was added to each sample collected in a 300 mL glass bottle. Assuming an initial sample volume of approximately 300 mL, this addition corresponded to a final formalin concentration of approximately 0.24% (v/v). The relatively dilute preservative was considered sufficient because the water samples contained comparatively less organic material and microbial biomass.

For the sediment samples, 15 mL of 10% formalin working solution was added to approximately 500 g of wet sediment. Because sediment is a solid heterogeneous matrix, a meaningful final volumetric formalin concentration could not be calculated; the preservative application is therefore reported as 15 mL of 10% formalin per 500 g wet sediment. The higher concentration was selected because sediment contained more organic matter and microbial biomass and had lower preservative penetration than the water samples. In both matrices, formalin was used to suppress microbial activity and minimize biological decomposition of organic material during temporary storage before MP extraction. Because the potential effects of formalin preservation on particle surfaces and polymer identification were not specifically evaluated, this procedure is acknowledged as a methodological limitation.

### 2.2. Laboratory Analysis

A total of 84 environmental samples (21 stations × two seasons × two matrices) were processed using procedures adapted from established MP protocols [31, 32]. Water samples were passed through 5 mm and 300 μm stainless‐steel sieves. Retained material was transferred to glass beakers and separated using 250 mL of saturated NaCl solution (*ρ* = 1.2 g cm^−3^). NaCl is accessible and widely used, but its density may provide incomplete recovery of high‐density polymers such as PET and PVC; the reported polymer composition should therefore be interpreted with this limitation in mind [33].

The samples were left undisturbed for 24 h to allow the buoyant MPs to separate, after which the supernatant was carefully collected. Organic matter was removed via oxidative digestion by adding 20 mL of 30% hydrogen peroxide (H_2_O_2_) and incubating the mixture for 24–72 h, following protocols that have been shown to improve MP extraction from organic‐rich matrices [34]. The resulting suspension was filtered onto Whatman No. 42 filter paper, and suspected MP particles were examined under a stereomicroscope (NZ1703‐P) equipped with Eye‐share software.

Sediment samples were processed separately following the procedure described by Masura et al. [31]. Approximately 500 g of wet sediment was oven‐dried at 90°C for 24 h, homogenized, and subsampled (100 g) for density separation using 250 mL of saturated NaCl solution (*ρ* = 1.2 g cm^−3^). After settling for 24 h, the supernatant was filtered and examined using the same stereomicroscopic procedure applied to water samples. The comparatively high drying temperature and the use of NaCl may have affected the recovery or properties of some polymers and are considered methodological limitations.

Suspected particles were classified visually as fragments, fibers, films, foams, or rounded particles. Rounded particles ≥ 300 μm were recorded as pellets for consistency with the original classification; however, smaller rounded particles may be described as spheres under other schemes [35]. Polymer identity was evaluated using ATR–FTIR (Agilent Cary 630, diamond crystal) for material from 16 of the 84 environmental samples (19%). Spectra were collected at 4 cm^−1^ resolution using 32 scans over 650–3000 cm^−1^ and compared with reference spectra. Polymer percentages reported below refer only to this analyzed subset and not to all visually identified particles.

A total of 44 visually identified particles were subjected to ATR–FTIR analysis [36]. Spectra were recorded at a resolution of 4 cm^−1^ using 32 scans over the 650–3000 cm^−1^ spectral range. The resulting spectra were compared with reference polymer spectra, including the FLOPP and FLOPP‐e libraries described by De Frond et al. [36]. A particle was accepted as plastic when its spectrum produced a hit‐quality index or spectral similarity of ≥ 70% with a reference polymer and its diagnostic absorption bands were consistent with the assigned polymer. All 44 analyzed particles met these identification criteria, corresponding to a confirmation rate of 100% among the spectroscopically examined particles. Because ATR–FTIR was conducted on a stratified subset rather than on every visually suspected particle, the resulting polymer composition represents only the 44 confirmed particles from the 16 selected samples and should not be extrapolated to the entire visually identified dataset.

### 2.3. Quality Assurance and Quality Control

Field and laboratory contamination was minimized by using precleaned glassware, aluminum‐foil covers, filtered reagents, cotton laboratory clothing, and thoroughly cleaned metal or glass equipment. One field blank and one laboratory blank were included during each of the two sampling campaigns, resulting in four procedural blanks. Field blanks were exposed during sample collection and handling. In contrast, laboratory blanks were subjected to the same preservation, density separation, digestion, filtration, and microscopic examination procedures applied to the environmental samples. All blanks contained ultrapure distilled water and were stored and processed alongside the corresponding environmental samples.

A total of 20 suspected MP particles, comprising 18 fibers and two fragments, were detected across the four procedural blanks. These blank particles were not subtracted from individual environmental sample counts because the blanks could not be normalized directly to the volume of water filtered by the Bongo net or the dry mass of sediment processed. The 20 particles represented approximately 0.11% of the 18,973 particles recorded from all environmental samples and therefore had a negligible influence on the overall abundance estimates. Nevertheless, because fibers accounted for 90% of the particles detected in the blanks, limited background contamination may have resulted in a slight overestimation of fiber abundance. The blank results were therefore used to evaluate the type and magnitude of potential contamination and are reported transparently rather than being used for numerical correction.

No polymer‐specific spike‐recovery or extraction‐efficiency experiment was conducted. Consequently, potential particle losses during sieving, density separation, transfer, digestion, and filtration could not be quantified. This limitation, together with the absence of sample‐specific blank correction, should be considered when interpreting the absolute MP abundance and the relative contribution of fibers.

### 2.4. Data Analysis

MP abundance and particle characteristics were summarized using Microsoft Excel and PAST v.5.2.2 [37]. Descriptive statistics are reported as means ± standard deviations, supplemented by medians and interquartile ranges for non‐normally distributed data. Data normality was evaluated using the Shapiro–Wilk and Anderson–Darling tests. Because the data did not satisfy the assumptions of parametric analysis, spatial differences in MP abundance among lake sectors were evaluated using the Kruskal–Wallis test. When a significant overall difference was detected, Dunn’s post hoc test was applied for pairwise comparisons. Seasonal differences between the dry and rainy seasons were evaluated separately for surface water and nearshore sediment using the appropriate nonparametric test. Statistical significance was set at *p* < 0.05. For each inferential analysis, the test statistic, sample size, exact *p*‐value, and adjusted post hoc *p*‐value are reported where applicable.

## 3. Results

### 3.1. Overview of Spatial and Seasonal Patterns

Across the 21 stations, surface‐water MP abundance was higher in the rainy season than in the dry season. In the northern sector, values ranged from 0.191 to 0.629 particles m^−3^ (0.364 ± 0.169) in the dry season and from 1.500 to 3.570 particles m^−3^ (2.334 ± 0.672) in the rainy season (Figure 2A,B). Corresponding dry‐ and rainy‐season means were 0.237 ± 0.038 and 1.461 ± 0.262 particles m^−3^ in the western sector, 0.267 ± 0.109 and 1.492 ± 0.303 particles m^−3^ in the eastern sector, and 0.269 ± 0.044 and 1.724 ± 0.305 particles m^−3^ in the southern sector.

**FIGURE 2:**
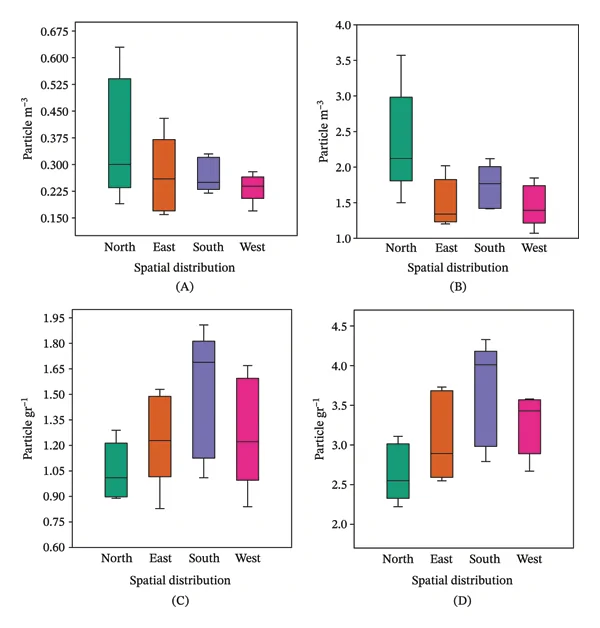
Spatial variation in MP abundance in Lake Toba: (A) surface water, rainy season; (B) surface water, dry season; (C) nearshore sediment, dry season; and (D) nearshore sediment, rainy season. Boxes show the distribution across stations; define the center line, box, whiskers, and any error metric used.

Nearshore sediment abundance was highest in the southern sector, ranging from 1.01 to 1.91 particles g^−1^ dry weight (1.514 ± 0.374) in the dry season and from 2.79 to 4.33 particles g^−1^ dry weight (3.666 ± 0.653) in the rainy season (Figure 2C,D). Northern‐sector means were 1.048 ± 0.160 and 2.628 ± 0.355 particles g^−1^ dry weight; eastern‐sector means were 1.248 ± 0.273 and 1.280 ± 0.326 particles g^−1^ dry weight; and western‐sector means were 3.088 ± 0.560 and 3.270 ± 0.385 particles g^−1^ dry weight in the dry and rainy seasons, respectively. The original analyses indicated no significant regional difference for the specified dry‐season comparisons (*p* > 0.05), whereas rainy‐season abundance varied among sectors (*p* = 0.025 for mp in water and 0.037 for mp in sediment), with northern and southern sectors differing from the other sectors.

MP abundance was consistently higher during the rainy season in both surface water and nearshore sediment (Figure 3A,B). Rainy‐season distributions also showed greater variability among stations, particularly in surface water. These observations describe seasonal differences in the sampled matrices; potential hydrological explanations are evaluated in the Discussion. The statistical analyses indicated seasonal differences in both matrices.

**FIGURE 3:**
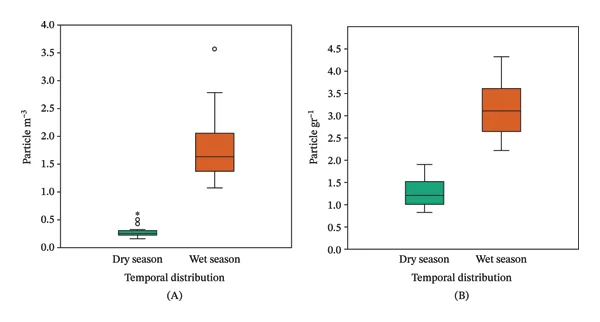
Seasonal variation in MP abundance in Lake Toba: (A) surface water and (B) nearshore sediment. Circles denote outliers, and asterisks denote extreme outliers. Define the center line, interquartile range, whiskers, and sample size in the final caption.

### 3.2. MP Characteristics

Four MP forms were recorded in surface‐water samples: fragments, fibers, films, and rounded particles classified as pellets. Detected particle sizes ranged from 300 to 5000 μm (Figure 4). Rounded particles occurred only during the rainy season and ranged from 300 to 2000 μm. Because particles near 300 μm may be classified as spheres rather than preproduction pellets, this category should be interpreted cautiously (Rochman et al. [35]). Overall, particles measuring 1000–2000 μm were the most frequently recorded size class. The reported size‐group comparison was not significant (*p* = 0.282), and comparisons among the other size categories were also not significant (*p* = 0.965).

**FIGURE 4:**
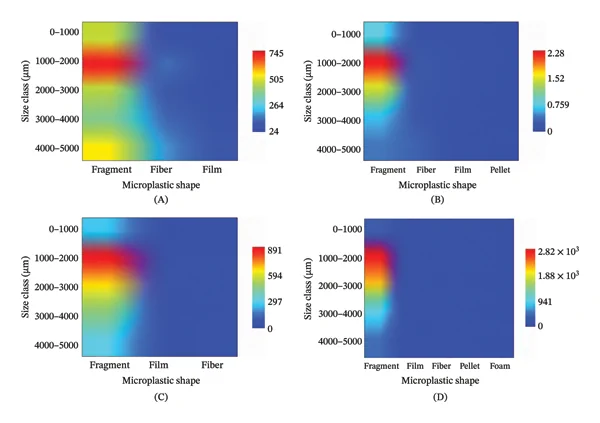
Distribution of MP counts by form and particle‐size class in (A) surface water during the rainy season, (B) surface water during the dry season, (C) nearshore sediment during the dry season, and (D) nearshore sediment during the rainy season. Warmer colors indicate higher counts; the same color scale is used across panels.

Five MP forms were recorded in nearshore sediments: fragments, fibers, films, rounded particles classified as pellets, and foams. The original dataset reported particles from 300 to 5000 μm while also assigning many fragments to the 300–1000 μm class. Particles measuring 1000–2000 μm were reported as the dominant class overall. Size‐class distributions did not differ significantly among sectors (*p* = 0.793) or between seasons (*p* = 0.290).

MP color varied between matrices and seasons (Figure 5). Black particles represented 43.29% of surface‐water MPs and 23.80% of sediment MPs, whereas blue particles represented 23.80% and 24.96%, respectively. Other colors included red, orange, yellow, purple, green, brown, transparent, and white. The original analysis indicated that black and blue differed from less frequent colors (*p* < 0.05).

**FIGURE 5:**
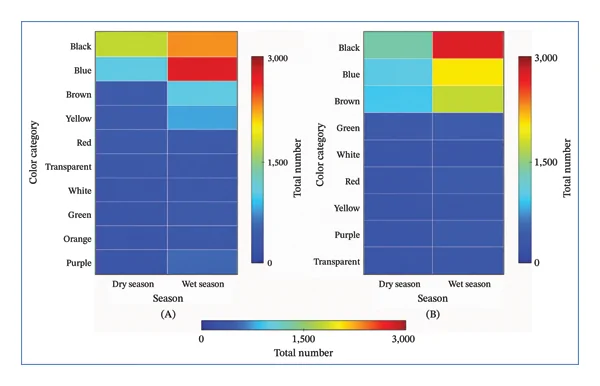
Color distribution of MPs in (A) surface water and (B) nearshore sediment during the dry and rainy seasons. Warmer colors indicate higher counts; the same color scale is used across panels.

Fragments were the dominant MP form in both matrices (Figure 6). Fragment abundance ranged from 0.269 to 2.500 particles m^−3^ in surface water (0.877 ± 0.576) and from 0.80 to 4.26 particles g^−1^ dry weight in sediment (2.109 ± 0.166). Fibers ranged from 0.025 to 0.896 particles m^−3^ (0.133 ± 0.161) in water and from 0 to 0.31 particles g^−1^ dry weight (0.034 ± 1.074) in sediment. Films, rounded particles, and foam occurred at lower abundances; foam was absent from water and occurred only at trace levels in sediment. The original analysis reported differences among MP forms (*p* < 0.05).

**FIGURE 6:**
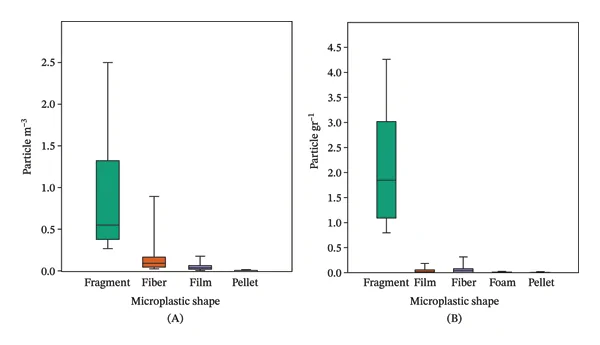
Abundance of MP forms in Lake Toba: (A) surface water and (B) nearshore sediment. Define all boxplot elements and sample sizes in the final caption.

Within the subset analyzed by ATR–FTIR, polyethylene (PE; 44.44%) and polypropylene (PP; 37.04%) were most frequent, followed by polyvinyl chloride (PVC; 7.41%), polyethylene terephthalate (PET; 7.41%), and polyamide (PA; 3.70%). These percentages characterize only the analyzed subset and should not be extrapolated to all visually identified particles. PE and PP are widely used in packaging and fisheries materials, so the composition is compatible with several potential catchment sources, but it does not establish source attribution. Furthermore, saturated NaCl may incompletely recover higher‐density polymers such as PET and PVC, potentially biasing their observed proportions downward.

## 4. Discussion

### 4.1. Lake‐Scale Distribution and Potential Sources

This study provides the first lake‐wide, seasonally resolved assessment integrating MPs in surface water and nearshore sediments of Lake Toba. The 21‐station design captured contrasting lake sectors, land uses, and hydrological settings during two seasons. Accordingly, the dataset establishes a baseline for the fractions and habitats sampled, while not representing MPs below approximately 300 μm, the vertical water column, or deep‐basin sediments. A total of 9734 suspected MP particles were identified in surface‐water samples from 16,207.86 m^3^ of filtered water, and 9239 particles were extracted from 4200 g of sediment. Fragments accounted for 80.23% of water MPs and 95.89% of sediment MPs, consistent with fragmentation of larger plastic items as an important pathway in freshwater systems (Tables 1 and 2). Degraded consumer items, fisheries and aquaculture materials, tourism‐related waste, and domestic litter are plausible contributors. However, these activities are treated as potential sources because no formal source‐apportionment analysis was performed.

**TABLE 1:** Microplastic abundance in surface water from various lakes worldwide.

No.	Location	Abundance	Dominant color	Dominant type	Size	Literature
1	Lake Singkarak, Indonesia	27.91 particles m^−3^	White and transparent	Fragment	300–1000 µm	[21]
2	Lakes Cibeureum, Cipondoh, Gede, Gong, Pluit, and Cincin, Indonesia	30,600 particles m^−3^	White	Foam	300–500 and 500–1000 µm	[23]
3	Lake Beratan, Indonesia	0.18–0.21 particles g^−1^	NR	Fragment	NR	[22]
4	Lake Towuti, Indonesia	7.36–26.28 particles L^−1^	Blue	Fragment	NR	[38]
5	Lake Chapala, Mexico	0.25–2.35 particles L^−1^	NR	NR	NR	
6	Lake Poyang, China	0.034 particles m^−3^	White	Fiber	0.1–0.5 mm	[39]
7	Lake Victoria, Africa	0.73 particles m^−3^	White and transparent	Fragment	NR	[40]
8	Lake Simcoe, Canada	0.04 particles L^−1^	Blue	Fiber	500–1000 µm	[41]
9	Patagonian lakes, Argentina	0.9 particles m^−3^	Blue	Fiber	≤ 1 mm	[42]
10	Lake Taihu, China	1.7–8.5 particles L^−1^	NR	Fragment	100–200 µm	[43]
11	Lake Chenghai, China	90–770 particles m^−3^	NR	Fiber	1–3 mm	[44]
12	Songshan Lake, China	2.29 ± 0.98 particles m^−3^	Transparent	Film	0.18–0.6 mm	[45]
13	Tangxun Lake, China	917.77 ± 742.17 particles m^−3^	NR	Fiber	NR	[46]
14	Semiremote boreal lakes, Canada	0.2 ± 0.3 particles L^−1^	NR	NR	NR	[47]
15	Lake Toba, Indonesia	0.629–3.570 particles m^−3^	Black	Fragment	1000–2000 µm	This study

*Note:* Values are presented in the units reported by the original studies.

Abbreviations: DW = dry weight, NR = not reported.

**TABLE 2:** Microplastic abundance in sediment from various lakes worldwide.

No.	Location	Abundance	Dominant color	Size range	Dominant type	Literature
1	Lake Beratan, Indonesia	0.01–2.53 particles g^−1^	NR	Fragment	< 5 mm	[22]
2	Lake Siombak, Indonesia	521–1481 particles kg^−1^	NR	Fragment	NR	[48]
3	Lake Poyang, China	11–3153 particles kg^−1^	NR	Fragment	< 2 mm	[49]
4	Lake Chapala, Mexico	210–219 particles kg^−1^	NR	NR	NR	[39]
5	Lake Taihu, China	460–1380 particles kg^−1^	White/transparent	Fragment	100–1000 µm	[43]
6	Lake Simcoe, Canada	8–1070 particles kg^−1^	Blue	Fragment	40–4270 µm	[41]
7	Lake Tollense, Germany	1410 ± 822 (lakeshore); 10,476 ± 4290 (lakebed) particles kg^−1^ DW	NR	NR	NR	[50]
8	Lake Superior, Canada	65 particles kg^−1^ DW	NR	Fragment	NR	[51]
9	Lake Chenghai, China	10–115 particles kg^−1^	NR	Fiber	< 1 mm	[44]
10	Songshan Lake, China	244 ± 121 particles kg^−1^	Transparent	Film	1–2 mm	[45]
11	Tangxun Lake, China	(1.81 ± 1.75) × 10^4^ particles kg^−1^	NR	Fragment	< 1 mm	[46]
12	Semiremote boreal lakes, Canada	551 ± 354 (shallow) and 177 ± 103 (deep) particles kg^−1^	NR	NR	NR	[47]
13	Lake Toba, Indonesia	0.83–4.33 particles g^−1^ DW	Black	Fragment	1000–2000 µm	This study

*Note:* Values are presented in the units reported by the original studies.

Abbreviations: DW = dry weight, NR = not reported.

Black particles were common, but color alone does not demonstrate enhanced sorption or ecological risk. Carbon‐black additives and environmental weathering can modify the sorptive behavior of some plastics [52, 53]. Because neither additive composition nor sorbed contaminants were measured here, any relationship between particle color and contaminant adsorption remains hypothetical.

Recent studies demonstrate that MP contamination occurs across lakes with markedly different climatic conditions, morphometry, catchment land use, and degrees of anthropogenic pressure. In Lake Toba, mean surface‐water abundance increased from 0.288 ± 0.110 particles m^−3^ in the dry season to 1.461 ± 0.836 particles m^−3^ in the rainy season. These concentrations were lower than those recently reported for Songshan Lake, China (2.29 ± 0.98 particles m^−3^), Lake Chenghai, China (90–770 particles m^−3^), and the nearshore waters of Tangxun Lake, China (917.77 ± 742.17 particles m^−3^) [44–46]. Considerably higher concentrations have also been reported for Lake Taihu (1.7–8.5 particles L^−1^) and Lake Chapala, Mexico (up to 2.35 particles L^−1^) [39, 54]. However, this apparent difference should not be interpreted directly as evidence that Lake Toba is less contaminated because the present study used a 300 μm Bongo net, whereas several recent studies used finer filters or direct water sampling and consequently included much smaller particles.

Sediment abundance in Lake Toba similarly increased from 1.26 ± 0.31 particles g^−1^ dry weight during the dry season to 3.14 ± 0.59 particles g^−1^ during the rainy season, equivalent to approximately 1260 and 3140 particles kg^−1^, respectively. These concentrations were higher than those recently reported for Songshan Lake (244 ± 121 particles kg^−1^), Lake Chenghai (10–115 particles kg^−1^), Lake Chapala (approximately 210–219 particles kg^−1^), and semiremote boreal lakes in Canada, where mean concentrations ranged from approximately 177 ± 103 particles kg^−1^ in deep sediments to 551 ± 354 particles kg^−1^ in shallow sediments [39, 44, 45, 47]. Conversely, sediment abundance in Lake Toba was lower than the mean of (1.81 ± 1.75) × 10^4^ particles kg^−1^ reported for the highly urbanized Tangxun Lake [46]. Thus, Lake Toba falls between less disturbed or moderately affected lakes and a strongly urbanized lake in terms of reported sediment abundance. This comparison should remain contextual because some studies reported concentrations per kilogram of wet sediment, whereas the present study used sediment dry weight.

The combination of relatively low surface‐water abundance and comparatively high sediment abundance in Lake Toba is consistent with the retention and settling of particles in bottom sediments. Recent investigations similarly identify lake sediments as important repositories of MPs. In Tangxun Lake, sediment contained substantially more particles than nearshore water, while MPs in Lake Taihu were detected throughout sediment profiles to a depth of 50 cm [43, 46]. In Lake Toba, the increased rainy‐season sediment abundance may reflect enhanced catchment input followed by settling, aggregation with organic or mineral particles, biofouling, or transport toward depositional areas. The higher sediment abundance in particular sectors may also be influenced by lake circulation, basin morphology, shoreline activities, and the redistribution of particles from shallow margins. These findings are consistent with sediment accumulation but do not independently demonstrate basin‐scale sediment focusing; confirmation would require sediment traps, hydrodynamic measurements, or dated sediment cores.

Fragments were the dominant morphology in both the water and sediment samples from Lake Toba, accounting for approximately 80.23% and 95.89% of the identified particles, respectively. This differs from recent observations in Lake Chenghai and semiremote Canadian boreal lakes, where fibers predominated, and from Songshan Lake, where films were commonly reported [44, 45]. Conversely, fragments were also among the dominant particle types in Tangxun and Taihu lakes [43, 46]. Such variation probably reflects differences in local sources and degradation processes. The prevalence of fragments in Lake Toba may indicate the breakdown of larger plastic items associated with packaging, household waste, tourism, fisheries, aquaculture, and shoreline activities. Nevertheless, particle morphology alone cannot identify a specific source because similar fragments may originate from multiple plastic products.

Within the ATR–FTIR‐analyzed subset, PE and PP were the most frequently identified polymers in Lake Toba. Their occurrence is consistent with recent studies from Songshan and Tangxun lakes, where these low‐density polymers were also commonly detected [46]. PE and PP are widely used in packaging, containers, ropes, fishing and aquaculture equipment, and other consumer products, making several local sources plausible. Although these polymers are initially buoyant, weathering, biofouling, aggregation with suspended material, and incorporation into fecal pellets may increase their effective density and promote deposition. However, because polymer identification in the present study covered material from only 16 of the 84 environmental samples, the reported polymer composition should be interpreted as descriptive of the analyzed subset and not as representative of all visually suspected particles.

### 4.2. Spatial Patterns and Possible Within‐Lake Redistribution

Surface‐water MP abundance was elevated in the northern sector (Figure 7), including stations near Haranggaol (Station 2) and Silalahi (Station 13), where floating net‐cage aquaculture is intensive. PP, polyester, and PA are used in aquaculture and fishing materials [55], making these activities plausible local contributors. Nevertheless, the ATR–FTIR subset and the absence of source‐specific tracers do not permit attribution of the observed MPs to aquaculture alone. Domestic waste, shoreline activity, river inputs, and tourism may also contribute.

**FIGURE 7:**
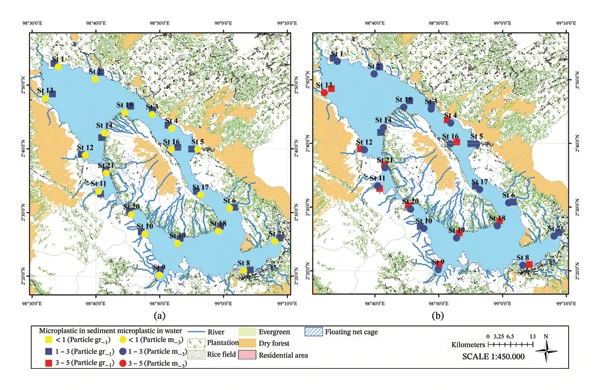
Distribution map of microplastics in the water and sediment of Lake Toba.

Nearshore sediment abundance was higher in the southern sector than in the northern sector. This spatial contrast may reflect differences in local inputs, shoreline retention, resuspension, particle density, biofouling, aggregation, or circulation. It is also compatible with redistribution toward lower‐energy depositional settings described in other lakes [44, 56]. However, because samples were collected only from nearshore sediments and no hydrodynamic or sediment‐transport measurements were made, downlake transport and basin‐scale sediment focusing cannot be distinguished from local accumulation.

Stations near more populated shorelines, including Pangururan (Station 21; 35,692 inhabitants) and Balige (Station 8; 46,585 inhabitants), tended to have higher MP abundance than less populated locations such as Lottung (3439 inhabitants) [57]. This pattern is consistent with studies linking freshwater MPs to population density, waste‐management limitations, and catchment runoff [50, 58, 59]. Population was not tested as an independent causal predictor in this study, so the association should not be interpreted as source confirmation.

### 4.3. Seasonal Variation and Management Implications

MP abundance was higher during the rainy season in both sampled matrices. Increased rainfall may increase river discharge and surface runoff, mobilizing terrestrial plastic debris, while seasonal winds may redistribute particles within the lake [44, 60]. These mechanisms are plausible explanations rather than direct observations because rainfall–runoff relationships, currents, and particle trajectories were not measured during the survey. The seasonal pattern observed in Lake Toba provides additional insight into possible transport processes. Surface‐water abundance was approximately five times higher during the rainy season than during the dry season. This pattern is consistent with enhanced delivery of land‐derived particles through surface runoff, tributary discharge, erosion, and the remobilization of plastic debris accumulated within the catchment. Increased rainfall may also transport particles from settlements, tourism facilities, agricultural areas, roads, and aquaculture zones into the lake. In contrast, a recent study in Lake Chapala recorded its highest water concentration during the low‐water period, with 2.35 particles L^−1^ in April compared with 1.8 particles L^−1^ during the rainy period [39]. The contrasting seasonal responses suggest that MP dynamics are lake‐specific. In shallow lakes, declining water levels and reduced dilution may concentrate particles during dry or low‐water periods, whereas in a deep tropical lake such as Lake Toba, rainfall‐driven catchment inputs may exert a stronger influence during the rainy season. Nevertheless, hydrological discharge and rainfall were not quantified concurrently in the present study; therefore, runoff should be regarded as a plausible explanation rather than a directly demonstrated transport mechanism.

Tourism centers such as Parapat and Tomok also showed elevated values at some stations. Consumption of single‐use plastics and limitations in waste collection may contribute to local inputs [61–63], but tourism and aquaculture contributions cannot be separated quantitatively with the present design. Management should therefore prioritize source inventories and routine monitoring around tourism centers, cage‐aquaculture areas, river mouths, populated shorelines, and reference sites. The occurrence of MPs in surface water and nearshore sediment is relevant to Lake Toba because the lake supports fisheries, tourism, and raw‐water supply. The present study did not evaluate drinking‐water exposure, biological uptake, toxicity, pathogens, or sorbed chemicals and therefore cannot quantify human health or ecological risk. A precautionary management response should combine improved solid‐waste collection, litter control at tourism and aquaculture facilities, runoff interception, and standardized monitoring across seasons and depths.

Taken together, the data show clear spatial and seasonal heterogeneity in the sampled fractions. They also identify a mismatch between sectors with elevated surface‐water abundance and those with elevated nearshore‐sediment abundance. This mismatch motivates, but does not prove, hypotheses concerning within‐lake redistribution and retention. Integrating these observations with hydrodynamic modeling and deep‐basin sampling is necessary before transport pathways or sediment focusing can be established. Future work should pair surface surveys with depth‐stratified water sampling, profundal sediment cores, current measurements, hydrodynamic modeling, and particle tracking. Finer analytical resolution is needed for MPs < 300 μm and nanoplastics, and spectroscopic confirmation should cover a larger, explicitly stratified subset or all suspected particles. Spike‐recovery experiments, more sensitive density solutions, standardized blank correction, contaminant analysis, and biological‐uptake studies would improve estimates of abundance, sources, exposure, and risk.

## 5. Conclusion

This study provides the first lake‐wide, two‐season baseline of MPs in the surface water and nearshore sediments of Lake Toba. MP abundance was higher during the rainy season in both matrices, fragments were dominant, and surface‐water and nearshore‐sediment maxima occurred in different lake sectors. These patterns are consistent with multiple local inputs and possible within‐lake redistribution, but they do not directly demonstrate source contributions, downlake transport, wind‐driven circulation, or sediment focusing. Management priorities include strengthened waste control around populated shorelines, tourism centers, river mouths, and cage‐aquaculture areas, together with standardized multidepth monitoring. Hydrodynamic measurements, deep‐basin sediments, broader spectroscopic confirmation, and explicit QA/QC correction are required to resolve the mechanisms and ecological implications.

## Author Contributions

Conception and design: Ahmad Muhtadi and Muhammad Reza Cordova. Data acquisition: Ahmad Muhtadi, Rusdi Leidonald, Prawira Atmaja Tampubolon, and Nur Rohim. Data analysis and interpretation: all authors. Original draft: Ahmad Muhtadi, Rusdi Leidonald, Prawira Atmaja Tampubolon, and Nur Rohim. Review and editing: Muhammad Reza Cordova and Mohd Yusoff Ishak.

## Funding

This study was supported by the RIIM LPDP Grant and the National Research and Innovation Agency (BRIN), 69/IV/KS/07/2024; Research Institute of Universitas Sumatera Utara, 206049.1/UN5.4.10. K/PPM/2024.

## Disclosure

All authors revised the manuscript and approved the final submitted version.

## Conflicts of Interest

The authors declare no conflicts of interest.

## Supporting Information

Additional supporting information can be found online in the Supporting Information section.

## Supporting information


**Supporting Information** Appendix 1. Sampling points and descriptions of the areas.

## Data Availability

The data that support the findings of this study are available from the corresponding author upon reasonable request.
