# Supplementary material for: Spatiotemporal Patterns of Microplastic Distribution in Surface Water and Nearshore Sediments of a Large Tropical Lake: Evidence From Lake Toba, Indonesia
Source: Scientifica (Cairo). 2026 Oct 28;2026:3525274. doi: 10.1155/sci5/3525274 (PMC13717233; doi:10.1155/sci5/3525274)
Supplement: Supplementary file 1 [file SCI5-2026-3525274-s001.docx]

Apendix 1. Sampling points and descriptions of the areas

| **Locations** | **Latitude** | **Longitude** | **Village** | **Regency** | **Description** |
| --- | --- | --- | --- | --- | --- |
| St1 | 2°53'47.42"N | 98°33'23.04"E | Bage | Karo | Village |
| St2 | 2°52'10.79"N | 98°40'34.84"E | Haranggaol | Simalungun | settlements, agriculture/ there is a river mouth, fishing activities, floating net cages (Floating net cage centered on Lake Toba), |
| St3 | 2°46'27.83"N | 98°48'45.64"E | Pematang Tambun Raya | Simalungun | Village, floating net cages (industry), fishing activities |
| St4 | 2°44'7.24"N | 98°51'42.36"E | Sipolha Horison | Simalungun | Village, forest |
| St5 | 2°40'4.70"N | 98°55'59.45"E | Parapat | Simalungun | settlements, hotel/tourism |
| St6 | 2°31'15.92"N | 99° 1'52.61"E | Hatinggian | Toba | Village, agriculture/ there is a river mouth |
| St7 | 2°25'38.01"N | 99° 8'25.57"E | Parparean | Toba | Outlet, fishing activities |
| St8 | 2°20'42.57"N | 99° 3'34.16"E | Balige | Toba | City of Toba Regency, hotel/tourism, fishing activities |
| St9 | 2°19'47.48"N | 98°49'35.96"E | Bakti Raja | Humbang Hasundutan | settlements, port, agriculture/ there is a river mouth; The largest river that flows into Lake Toba |
| St10 | 2°26'25.76"N | 98°47'5.66"E | Sabulan | Samosir | Village, port |
| St11 | 2°33'25.31"N | 98°40'3.70"E | Turpuk Sihotang | Samosir | settlements, hotel/tourism, fishing activities |
| St12 | 2°38'47.99"N | 98°37'5.66"E | Bonan Dolok | Samosir | Village, agriculture/ there is a river mouth, fishing activities |
| St13 | 2°48'19.66"N | 98°31'46.17"E | Silalahi | Dairi | settlements, agriculture/ there is a river mouth, fishing activities, floating net cages (Floating net cages centered on Lake Toba, besides haranggaol), livestock |
| St14 | 2°41'15.83"N | 98°41'10.89"E | Situngkir | Samosir | Village, agriculture/ there is a river mouth, fishing activities |
| St15 | 2°45'16.34"N | 98°44'37.66"E | Simanindo | Samosir | hotel/tourism, Port (The main port in Samosir) |
| St16 | 2°39'41.70"N | 98°51'18.58"E | Tomok | Samosir | Village, hotel/tourism, |
| St17 | 2°33'29.70"N | 98°54'56.77"E | Silimalombu | Samosir | Village, floating net cages (industry) |
| St18 | 2°27'52.12"N | 98°59'45.05"E | Onan Runggu | Samosir | Village |
| St19 | 2°25'57.46"N | 98°54'13.23"E | Nainggolan | Samosir | Village, port, fishing activities |
| St20 | 2°30'12.36"N | 98°45'55.84"E | Tarabung | Samosir | Agriculture; there is a river mouth |
| St21 | 2°36'17.93"N | 98°41'35.75"E | Pangururan | Samosir | City of Samosir Regency, hotel/tourism, fishing activities |
